# Effects of Shenmai injection combined with platinum-containing first-line chemotherapy on quality of life, immune function and prognosis of patients with nonsmall cell lung cancer: A protocol for systematic review and meta-analysis

**DOI:** 10.1097/MD.0000000000027524

**Published:** 2021-11-05

**Authors:** Yanqiong Chen, Chao Zhang, Cheng Pan, Yunkui Yang, Jin Liu, Jialing Lv, Guilin Pan

**Affiliations:** a Qujing No. 1 Hospital, Qujing, Yunnan Province, China; b Kunming Medical University, Kunming, Yunnan Province, China.

**Keywords:** meta-analysis, nonsmall cell lung cancer, platinum, protocol, Shenmai injection

## Abstract

**Background:**

Lung cancer is the leading cause of death among cancer patients worldwide. Close to 85% of lung cancer pathology types are nonsmall cell lung cancer (NSCLC). With advances in medicine, the survival rate of early-stage NSCLC has improved. Nevertheless, about 70% of patients are diagnosed at an advanced stage, and chemotherapy is the primary treatment option. Chemotherapy causes toxic side effects such as bone marrow suppression, gastrointestinal reactions, and damage to vital organs, which are difficult for patients to tolerate. Many published literatures have reported that Shenmai injection (SMI) combined with platinum-containing first-line chemotherapy regimen for NSCLC can improve the recent efficacy, reduce toxic side effects and improve the quality of life. However, most of the studies were small samples and lacked persuasive power, while controversies existed among individual studies. Therefore, this study used meta-analysis to further evaluate the effects of SMI combined with platinum-containing first-line chemotherapy on the quality of life, immune function and prognosis of patients with NSCLC.

**Methods:**

Wanfang, Chinese Biomedical Literature Database, Chinese National Knowledge Infrastructure, the Chongqing VIP Chinese Science and Technology Periodical Database, PubMed, Embase, and Web of Science databases were searched. The search was scheduled from the establishment of the database to September 2021. All randomized controlled trials comparing SMI in combination with platinum-containing first-line chemotherapy to platinum-containing first-line chemotherapy alone for the treatment of NSCLC were searched and evaluated for inclusion. Two investigators independently performed study selection, data extraction and synthesis. The Cochrane Risk of Bias tool was used to assess the risk of bias in the randomized controlled trials. Stata 16.0 software was used for meta-analysis.

**Results:**

The results of this meta-analysis will be submitted to a peer-reviewed journal for publication.

**Conclusion:**

This study comprehensively evaluated the effects of SMI combined with platinum-containing first-line chemotherapy on quality of life, immune function and prognosis in patients with NSCLC to provide an evidence-based basis for clinical practice.

**Ethics and dissemination:**

The private information from individuals will not be published. This systematic review should also not damage participants’ rights. Ethical approval was not available. The results may be published in a peer-reviewed journal or disseminated in relevant conferences.

**OSF Registration number:** DOI 10.17605/OSF.IO/AMKDC

## 1 Introduction

Lung cancer is one of the leading causes of cancer-related deaths worldwide.^[1]^ In 2018, there were an estimated 2.1 million new cases of lung cancer and 1.8 million deaths worldwide, accounting for 11.6% of cancer incidence and 18.4% of deaths, respectively.^[2]^ It is estimated that within 5 years, the number of patients diagnosed with lung cancer worldwide will reach 2.13 million. Eighty-five percent of lung cancers are nonsmall cell lung cancer (NSCLC).^[2]^ The majority of the patients are diagnosed with advanced stage and have a poor prognosis.

Currently, surgery is still the mainstay of early treatment for NSCLC; however, about 70% of patients are diagnosed at an advanced stage and chemotherapy is the main treatment option.^[3]^ Although chemotherapy has significant efficacy in reducing tumor size, it is often accompanied by severe toxic side effects that are difficult for patients to tolerate.^[4]^ Therefore, the search for potent and toxic reducing drugs has become one of the relevant research directions.

At present, traditional Chinese medicine (TCM) has become a common method for clinical treatment of NSCLC.^[5]^ Some studies have confirmed that the combination of Chinese medicine and chemotherapy can improve the clinical treatment effect, achieve the effect of increasing efficiency and reducing toxicity, significantly reduce the adverse reactions caused by chemotherapy, improve the tolerance of chemotherapy, and improve the quality of life of patients.^[6–8]^

Shenmai injection (SMI) is a pure TCM preparation, consisting of ophiopogonis radix and ginseng radix rubra, whose active ingredients include panaxan, ginsenoside, ophiopogonin, ophiopogonanone, and ophiopogon polysaccharides.^[9,10]^ SMI has the effects of activating the body's immune system, improving the hematopoietic function of bone marrow, regulating angiogenesis and inhibiting the growth of tumor cells.^[11–13]^ Modern pharmacological research proves that ginsenosides have biphasic immunomodulatory effects and can improve the nonspecific immunity and specific immune function of the body.^[10,14]^ Ophiopogon polysaccharides has a promoting effect on humoral immunity and cellular immunity, and can induce a variety of cytokines.^[15]^ Therefore, SMI can significantly improve the immune function of tumor patients, and has a certain synergistic effect when combined with chemotherapeutic drugs, and can effectively reduce the toxic side effects caused by chemotherapeutic drugs.^[16–18]^

Many published literatures now report that SMI combined with platinum-containing first-line chemotherapy for NSCLC can improve near-term efficacy, reduce toxic side effects, and improve the quality of life.^[19–23]^ However, most of the studies were small samples and lacked persuasive power, while controversies existed among individual studies. Therefore, this study used meta-analysis to further evaluate the effects of SMI combined with platinum-containing first-line chemotherapy on quality of life, immune function and prognosis of NSCLC patients.

## 2 Methods

### 2.1 Study registration

The protocol of the systematic review has been registered on Open Science Framework. The registration number was DOI 10.17605/OSF.IO/AMKDC. This meta-analysis protocol was based on the Preferred Reporting Items for Systematic Reviews and Meta-analysis Protocols Statement Guidelines.^[24]^

### 2.2 Inclusion criteria

#### 2.2.1 Types of studies

(1) Randomized controlled trials (RCTs), with no language restriction; and
(2) Studies dealing with the effect of SMI combined with platinum-containing first-line chemotherapy on quality of life, immune function, and prognosis of NSCLC patients.

#### 2.2.2 Types of participants

Patients with a clear diagnosis of stage III/IV NSCLC, the age, sex, and ethnicity were not limited.

#### 2.2.3 Types of interventions

The experimental groups were treated with SMI combined with platinum-containing first-line chemotherapy, while the control groups were treated with platinum-containing first-line chemotherapy alone.

#### 2.2.4 Types of outcome measures

(1) The primary outcomes: disease-free survival (DFS) and overall survival (OS).
(2) The secondary outcomes: quality of life: scores of various quality of life scales; and immune function: including assessment of T-lymphocytes, CD3+, CD4+, and CD8+; and
(3) Adverse events: including assessment of changes in white blood cells, hemoglobin, platelets, renal function, liver function, and incidence of nausea and emesis.

### 2.3 Exclusion criteria

(1) Studies with duplicate results;
(2) Animal experiments;
(3) Studies with the absence of complete data or full text literature; and
(4) Non-RCTs.

### 2.4 Search methods for the identification of studies

#### 2.4.1 Search strategy

In this systematic review and meta-analysis, we searched the database of Wanfang, Chinese Biomedical Literature Database, Chinese National Knowledge Infrastructure, the Chongqing VIP Chinese Science and Technology Periodical Database, PubMed, Embase, and Web of Science to identify all eligible studies. All searches were performed in September 2021. The search strategy for PubMed is exhibited in Table 1. The retrieval strategy of other electronic databases was performed on the basis of PubMed. According to the characteristics of each database, the retrieval strategy could be changed slightly.

**Table 1:** Search strategy in PubMed database.

Number	Search terms
#1	Carcinoma, Nonsmall-Cell Lung [MeSH]
#2	Carcinoma, Nonsmall Cell Lung [Title/Abstract]
#3	Nonsmall Cell Lung Cancer [Title/Abstract]
#4	Nonsmall-Cell Lung Carcinoma [Title/Abstract]
#5	Nonsmall Cell Lung Cancer [Title/Abstract]
#6	Carcinoma, Nonsmall Cell Lung [Title/Abstract]
#7	Carcinomas, Nonsmall-Cell Lung [Title/Abstract]
#8	Lung Carcinoma, Nonsmall-Cell [Title/Abstract]
#9	Lung Carcinomas, Nonsmall-Cell [Title/Abstract]
#10	Nonsmall Cell Lung Carcinoma [Title/Abstract]
#11	Nonsmall-Cell Lung Carcinomas [Title/Abstract]
#12	OR/1-11
#13	Platinum [MeSH]
#14	Platinum Black [Title/Abstract]
#15	Cisplatin [MeSH]
#16	Platinum Diamminodichloride [Title/Abstract]
#17	cis-Diamminedichloroplatinum(II )[Title/Abstract]
#18	cis-Dichlorodiammineplatinum(II) [Title/Abstract]
#19	Biocisplatinum [Title/Abstract]
#20	Dichlorodiammineplatinum [Title/Abstract]
#21	NSC-119875 [Title/Abstract]
#22	Platidiam [Title/Abstract]
#23	Platino [Title/Abstract]
#24	Platinol [Title/Abstract]
#25	cis-Diamminedichloroplatinum [Title/Abstract]
#26	cis-Platinum [Title/Abstract]
#27	Diamminodichloride, Platinum [Title/Abstract]
#28	cis Diamminedichloroplatinum [Title/Abstract]
#29	cis Platinum [Title/Abstract]
#30	Carboplatin [MeSH]
#31	cis-Diammine(cyclobutanedicarboxylato)platinum II [Title/Abstract]
#32	Almirall Brand of Carboplatin [Title/Abstract]
#33	Blastocarb [Title/Abstract]
#34	Bristol-Myers Squibb Brand of Carboplatin [Title/Abstract]
#35	CBDCA [Title/Abstract]
#36	Carboplat [Title/Abstract]
#37	Carbosin [Title/Abstract]
#38	Carbotec [Title/Abstract]
#39	Chiesi Brand of Carboplatin [Title/Abstract]
#40	Columbia Brand of Carboplatin [Title/Abstract]
#41	Ercar [Title/Abstract]
#42	JM-8 [Title/Abstract]
#43	Lemery Brand of Carboplatin [Title/Abstract]
#44	NSC-241240 [Title/Abstract]
#45	Nealorin [Title/Abstract]
#46	Neocarbo [Title/Abstract]
#47	Neocorp Brand of Carboplatin[Title/Abstract]
#48	Paraplatin [Title/Abstract]
#49	Paraplatine [Title/Abstract]
#50	Pharmachemie Brand of Carboplatin [Title/Abstract]
#51	Platinwas [Title/Abstract]
#52	Prasfarma Brand of Carboplatin [Title/Abstract]
#53	Ribocarbo [Title/Abstract]
#54	ribosepharm Brand of Carboplatin [Title/Abstract]
#55	JM 8 [Title/Abstract]
#56	JM8 [Title/Abstract]
#57	NSC 241240 [Title/Abstract]
#58	NSC241240 [Title/Abstract]
#59	OR/13-58
#60	Shenmai Injection [Title/Abstract]
#61	Ginseng Injection [Title/Abstract]
#62	OR/60-61
#63	Randomized Controlled Trials as Topic [MeSH]
#64	Clinical Trials, Randomized [Title/Abstract]
#65	Controlled Clinical Trials, Randomized [Title/Abstract]
#66	Trials, Randomized Clinical [Title/Abstract]
#67	Random^∗^ [Title/Abstract]
#68	OR/63-67
#69	#12 and #59 and #62 and #68

#### 2.4.2 Study selection

**Figure 1:**
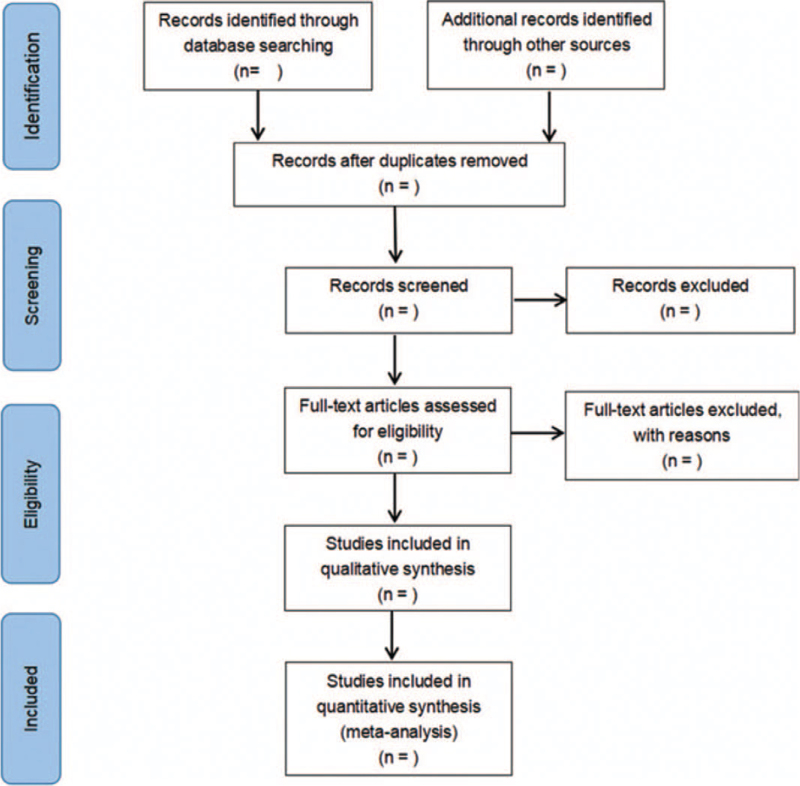
Flow diagram of study selection process.

The literature screening process is illustrated in Figure 1. According to the literature inclusion and exclusion criteria, the retrieval results were screened strictly by 2 researchers, and when there were differences, they were judged by discussion or by a third party.

#### 2.4.3 Data collection and management

Two investigators independently abstracted the data on the studies. Discrepancies were resolved by consensus, referring back to the original study, or consulting a third reviewer. Data extraction contents included: the basic characteristics of the study, including the first author, the year of publication, the country of publication, the source of the study population, the number of cases, indicators of observation, name, dosage, components, frequency of application, and course of treatment of drugs; the required data from the survival curve obtained by extracting directly from the article or using Engauge Digitizer 4.1 software (http://digitizer.sourceforge.net/), which was then used to obtain the hazard ratio of OS and DFS through calculation, corresponding to the 95% confidence intervals (CIs), for prognosis analysis; and clinicopathological data, including age, sex, tumor size, TNM stage, differentiation, lymphatic metastasis, and so on.

### 2.5 Assessment of risk of bias in included studies

Two evaluators independently evaluated the quality of the included RCTs with the Cochrane Risk Assessment Manual.^[25]^ The evaluation results were classified into the high-risk, low-risk, and unclear categories.

### 2.6 Measurement of treatment effect

For dichotomous data, the risk ratio and its 95% CIs would be shown. For continuous data, the standardized mean difference of its 95% CIs would be presented. OS and DFS were taken as prognostic outcomes. The results were expressed as hazard ratios, with 95% CIs.

### 2.7 Management of missing data

If there existed insufficient or missing data in the literatures, we would just analyze the currently available data and discuss its potential value.

### 2.8 Statistical analysis

STATA 16.0 (STATA Corporation, College Station, TX) was used for this meta-analysis. The chi-square test was used to test the heterogeneity of the included studies. If *P* ≥ .1, *I*^*2*^ ≤ 50%, there was no statistical heterogeneity among the results of the studies, and a fixed-effect model (Mantel–Haenszel method) was used for analysis; otherwise, a random-effect model was used.

### 2.9 Additional analysis

#### 2.9.1 Subgroup analysis

We conducted a subgroup analysis based on ethnicity and survival data sources.

#### 2.9.2 Sensitivity analysis

The sensitivity analysis was conducted by adopting the approach of excluding the studies one by one.

#### 2.9.3 Publication bias

Egger test was performed to evaluate publication bias, and calculate *t* value and *P* value, with *P* < .05.^[26,27]^ It was considered that there existed publication bias.

### 2.10 Ethics

Our research data were derived from published literatures, since there were no patient recruitment and personal information collection. Therefore, ethical approval was not required.

## 3 Discussion

TCM considered lung cancer to be a disease with excess syndrome caused by deficiency, and the main treatment principle should be to benefit Qi and nourish Yin.^[28]^ Chemotherapy alone further damages the patient's positive Qi and therefore reduces the immune capacity. Therefore, TCM believes that tumor treatment should insist on tonifying Qi and blood and supporting positive Qi.^[29]^

SMI is extracted from 2 herbs, ophiopogonis radix and ginseng radix rubra.^[30,31]^ Ginseng radix rubra has the traditional effects of tonifying the vital energy, nourishing the spleen, benefiting the lung, nourishing the body and quenching thirst, and calming the mind.^[32]^ Ophiopogonis radix has the function of moistening the lung and nourishing the yin, benefiting the stomach and generating fluid, clearing the heart and removing irritation, and laxing the bowels.^[33]^ Both herbs belong to the lung meridian, and their combination has the effects of moistening the lung, nourishing yin and the qi, and nourishing the pulse. Modern pharmacological studies have demonstrated that SMI has the ability to regulate immune function, enhance body immunity, be antitumor, reduce the toxic side effects of chemotherapy and increase the tolerance of patients to chemotherapy drugs.^[34–36]^ This study will comprehensively evaluate the effects of SMI combined with platinum-containing first-line chemotherapy on the quality of life, immune function and prognosis of patients with nonsmall cell lung cancer, which will provide an evidence-based basis for clinical practice.

## Author contributions

**Conceptualization:** Yanqiong Chen, Guilin Pan.

**Data curation:** Yanqiong Chen, Chao Zhang.

**Formal analysis:** Yanqiong Chen, Chao Zhang.

**Funding acquisition:** Guilin Pan.

**Investigation:** Chao Zhang, Cheng Pan.

**Methodology:** Chao Zhang, Cheng Pan.

**Project administration:** Guilin Pan.

**Resources:** Cheng Pan, Yunkui Yang.

**Software:** Yunkui Yang, Jin Liu.

**Supervision:** Guilin Pan.

**Validation:** Jin Liu, Jialing Lv.

**Visualization:** Jin Liu, Jialing Lv.

**Writing – original draft:** Yanqiong Chen, Guilin Pan.

**Writing – review & editing:** Yanqiong Chen, Guilin Pan.
